# Integrating 3D technology with the Sampaio classification for enhanced percutaneous nephrolithotomy in complex renal calculi treatment

**DOI:** 10.3389/fsurg.2024.1471958

**Published:** 2024-10-22

**Authors:** Jiamo Zhang, Jing Qing, Ke Hu, Honglin Cheng

**Affiliations:** ^1^ Department of Urology, Yangchuan Hospital, Chongqing Medical University, Chongqing, China; ^2^ Department of Urology, The First Affiliated Hospital of Chongqing Medical University, Chongqing, China

**Keywords:** complex renal calculi, three-dimensional reconstruction, pelvicalyceal system, percutaneous nephrolithotomy, stone clearance

## Abstract

**Background:**

To investigate the safety and efficacy of percutaneous nephrolithotomy (PCNL) in the treatment of complicated renal calculi by integrating three-dimensional (3D) computed tomography (CT) reconstruction with the Sampaio classification of the renal collecting system.

**Methods:**

Sixty-four consecutive patients with complex kidney calculi who underwent PCNL between January 2019 and October 2023 were retrospectively analyzed and divided into experimental group (3D printing) and control group (CT imaging) according to their willingness to pay for 3D imaging. Both groups underwent preoperative CT urography. The Digital Imaging and Communications (DICOM) in Medicine data of the experimental group from CT imaging were used for 3D reconstruction and model printing. Then, the Sampaio classification system was used to design the puncture channel and develop a surgical strategy.

**Results:**

The 3D-printed models of the experimental group successfully displayed the Sampaio classification system. There was no significant difference in the baseline parameters between the groups. Compared with the control group, the experimental group exhibited significant improvements in the puncture time, number of puncture needles, number of puncture channels, target calyx consistency, number of first puncture channels, and stone clearance. There were no significant differences in the total operative time, decrease in the hemoglobin level, length of hospital stay, and postoperative complications between the groups.

**Conclusions:**

Integration of 3D technology with the Sampaio classification of the renal collecting system can enhance the preoperative evaluation and planning of percutaneous renal access. This approach allows a more precise method of PCNL for treating complex renal calculi.

## 1 Introduction

Percutaneous nephrolithotomy (PCNL) is considered the standard of care for the treatment of complex renal calculi (1), which are characterized by intricate renal pelvic anatomy and large stone burdens in the renal collecting system or multiple renal calyces. Because the use of multiple puncture channels to enhance stone clearance is often necessary, unwarranted punctures are prone to serious perioperative complications, such as bleeding and damage to adjacent organs (2). Therefore, the strategic design and precise creation of appropriate puncture channels are crucial for improving stone clearance and reducing complications (3). A thorough understanding of the three-dimensional (3D) relationship between intrarenal calculi and the renal collecting system is vital for planning and establishing the optimal access to the pelvicalyceal system. Researchers are continuously exploring new and enhanced methods to assist and refine PCNL to achieve higher stone clearance rates and fewer complications (4). In recent years, 3D printing technology has resulted in remarkable improvements in clinical medicine, tissue engineering, medical devices, and anatomical models (5). Additionally, 3D printing technology has been extensively used in the field of urology preoperative planning of renal and prostate cancers (6), with a particular focus on complex renal calculi as an important area of research (7, 8). Although most studies have focused on adjacent perirenal structures, there is a lack of literature regarding the integration of 3D technology with renal pelvis and calyces classification theories. This study investigated the use of 3D technology in conjunction with the Sampaio collecting system classification theory to enhance preoperative planning and assess the safety and efficacy of PCNL.

## 2 Materials and methods

This retrospective study was conducted in accordance with the Strengthening the Reporting of Observational studies in Epidemiology (STROBE) Statement and included 64 consecutive patients with complex renal calculi treated with PCNL between January 2019 and October 2023. Patients who had a history of mononephrous and open stone surgery, coagulation abnormalities, cardiorespiratory abnormalities, and other contraindications to surgery were excluded. The participants were divided into the experimental group (3D printing; *n* = 32) and the control group [computed tomography (CT) imaging; *n* = 32] based on the use of 3D reconstruction models. The CT image Digital Imaging and Communications in Medicine (DICOM) data of the experimental group were used for 3D reconstruction and model printing that incorporated the Sampaio classification of the renal collecting system to design the puncture channel and formulate a surgical plan. However, for the control group, the puncture channel was designed on the basis of two-dimensional CT images. Preoperative demographic characteristics and specific parameters, such as sex, age, body mass index, S.T.O.N.E. renal calculus scores (9), underlying diseases, preoperative creatinine levels, urine culture results, and American Society of Anesthesiologists (ASA) scores of both groups were recorded separately. This study adhered to the Declaration of Helsinki and was approved by the Ethics Committee of Yongchuan Hospital Affiliated to Chongqing Medical University (approval no. 165). Signed informed consent was obtained preoperatively from patients and their family members in both groups.

### 2.1 Production of 3D models

#### 2.1.1 Ct urography protocol

A 64-multidetector row CT scanner (Siemens, Munich, Germany) was used to acquire plain scan, arterial phase, venous phase, and excretory phase data of the kidneys at 1-mm step intervals. Nonionic contrast medium was injected at a dose of 1.5–2 mL/kg through the anterior elbow artery (total of 80–100 mL) at a rate of 2.0–2.5 mL/s. Subsequent continuous scanning was performed with delays of 25, 60, and 600–900 seconds after injection.

#### 2.1.2 Image postprocessing

The DICOM data of the CT images were extracted and imported into Mimics software (version 18.0; Materialise, Leuven, Belgium; https://www.materialise.com) for postprocessing, which included tasks such as image segmentation, 3D reconstruction, adjustment, and supplementation. To obtain 3D-reconstructed images of various sections of the kidney, a combination of threshold selection, region growth, multilayer editing, and modification techniques were used. Then, these images were merged to generate a comprehensive kidney model in the Standard Template Library (STL) format. An experienced urologist and radiologist performed the entire procedure.

#### 2.1.3 3D model printing

The 3D models in STL format were printed using a 3D printer (model iSLA450; Shining 3D Technology Co., Ltd., Hangzhou, China). Photosensitive resin was used as the raw material, and the kidney parenchyma was made of a soft transparent material. Different colors of transparent resin material were automatically added by the 3D printer to create a multicolored model. The renal artery appeared red, renal vein had a blue hue, renal collecting system was a light color, and kidney calculi were a darker color.

### 2.2 Preoperative design and surgical methods

#### 2.2.1 Preoperative design planning

The 3D-printed model integrated with the Sampaio classification system was used for the experimental group (10). However, two-dimensional CT images were used for the control group. Preoperative planning was performed for both groups to identify the target calyx for the puncture, determine the number and sequence of puncture channels, and predict stone clearance. The experimental group can perform simulated puncture and training using 3D-printed models.

#### 2.2.2 Surgical methods

The patients underwent retrograde insertion of a 6-Fr ureteral catheter into the renal pelvis while in the prone split-leg position. The intraoperative ultrasound-guided renal calyceal puncture was performed on the basis of the preoperative planning for both groups. The puncture site was typically identified between the 11th and 12th intercostal spaces, located between the posterior axillary line and the subscapularis angle line. The ultrasound probe and puncture needle were aligned in the same plane to approach the target calyx through the renal papilla. After the insertion of a J-shaped guidewire in the calyx, a fascial dilator was used to progressively expand the channel, resulting in a working channel of 20-Fr to 24-Fr. Pneumatic ballistic lithotripsy and ultrasound (Electro Medical Systems, Nyon, Switzerland) were used to fragment the stone. Additional channels were established as needed, and a 6-Fr D-J tube and 16-Fr nephrostomy tube were inserted postoperatively. CT images were obtained 1–2 days after surgery to assess stone retention and the position of the double-J ureteral stent.

### 2.3 Observation index

#### 2.3.1 Intraoperative

This study analyzed the following key factors: time required to localize the puncture (from the start of ultrasound scanning to guidewire entry in the renal pelvis), consistency between the intraoperative actual target calyx and the preoperative planned target calyx, number of puncture channels used, number of puncture needles established in the first channel, and total operative time (from anesthesia induction to extubation).

#### 2.3.2 Postoperative

The stone-free rate was defined as the absence of stone particles larger than 2 mm, postoperative complications assessed on the basis of the Clavien-Dindo classification (11), decrease in hemoglobin, and length of hospital stay.

### 2.4 Statistical analysis

Statistical analyses were performed using SPSS version 25.0 (IBM, Chicago, IL, USA). Continuous data were reported as the mean ± standard deviation for normally distributed data and as the median (interquartile range) for non-normally distributed data. Student's *t*-test was performed for normally distributed data, whereas the Wilcoxon rank-sum test was performed for non-normally distributed data. Categorical data were assessed using the chi-squared test or Fisher's exact test. A *p*-value <0.05 was considered statistically significant.

## 3 Results

### 3.1 Patient characteristics

**Figure 1:**
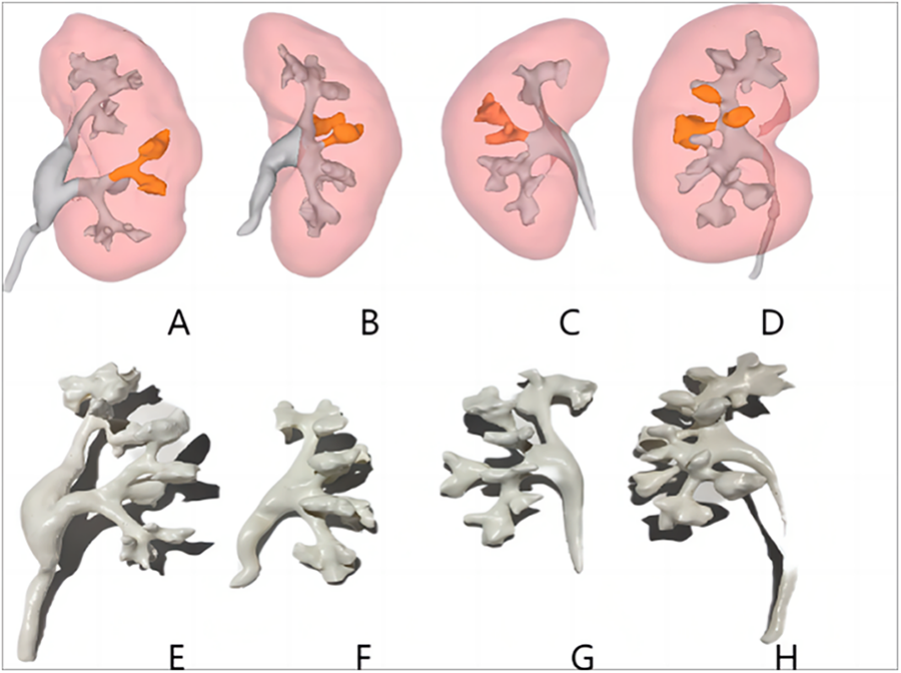
Three-dimensional reconstructed images of each type of Sampaio classification of the renal collecting system **(A–D)** and three-dimensional printed models **(E–H)**. **(A,E)** Type A-I collecting system in which the calyces in the middle part of the kidney are subordinate to the major calyces in the lower kidney pole. **(B,F)** Type A-II collecting system in which the calyces in the middle of the kidney are subordinate to the major calyces at the upper and lower kidney poles, respectively, with a calyx–neck crossover. **(C,G)** Type B-I collecting system with a separate major calyx in the middle of the kidney draining urine into the renal pelvis. **(D,H)** Type B-II collecting system, the central part of the kidney is drained directly into the renal pelvis by 2–4 minor calyces.

**Figure 2:**
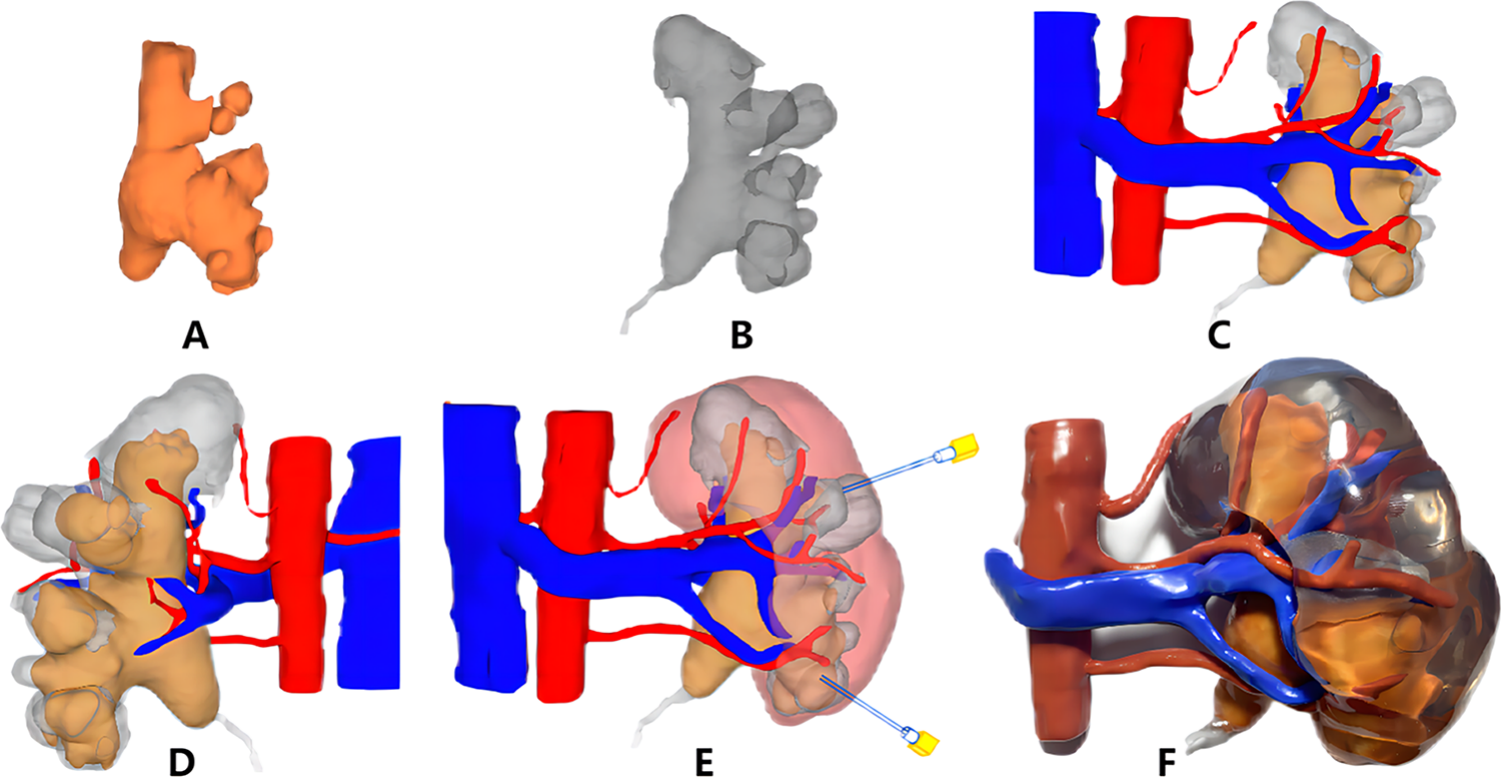
Three-dimensional (3D) digital reconstruction **(A–E)** and 3D printing of the kidney model **(F)**. **(A)** Stone; **(B)** collection system; **(C)** front of the collection system, stones, and vasculature; **(D)** back of the collection system, stones, and vasculature; **(E)** preoperative puncture access planning; **(F)** 3D printing kidney model and stones.

There were no significant differences in sex, age, body mass index, S.T.O.N.E. score, hypertension, diabetes mellitus, creatinine status, and American Society of Anesthesiologists classification of the two groups (*p* > 0.05) (Table 1). Successful completion of 3D reconstruction and 3D printing of both the affected and normal kidneys was achieved for all 32 patients in the experimental group. 3D reconstruction effectively displayed the shapes of the renal pelvis and calyces, as well as the size and distribution of the stones and renal vascularization. Furthermore, classification based on the Sampaio collecting system was performed (Figure 1), and a comprehensive analysis of the stones on the basis of the Sampaio classification system was conducted to enable preoperative planning and designing of the puncture channel (Figure 2).

**Table 1:** Patient characteristics.

Characteristics	Experimental group (*n* = 32)	Control group (*n* = 32)	*p*-value
Sex (female/male)	17/15	18/14	0.802
Age (years)	56.81 (±7.05)	58.44 (±7.55)	0.377
BMI^a^ (kg/m^2^)	22.35 (±3.29)	23.59 (±2.86)	0.113
S.T.O.N.E. score	8.81 (±1.20)	8.75 (±1.44)	0.851
Hypertension (%)	34.38%	31.25%	0.79
Diabetes (%)	21.88%	18.75%	0.756
Creatinine (µmol/L)	84.75 (20.79)	88.91 (30.90)	0.530
Urine culture (+)	28.13%	15.63%	0.226
ASA^a^ score (*n*)			0.888
1	19	18	
2	12	14	
3	1	0	
4	0	0	

^a^ BMI, body mass index; ASA, American Society of Anesthesiologists.

### 3.2 Perioperative data

Both groups underwent PCNL without serious complications, such as vascular and organ damage or sepsis. During PCNL, compared with the control group, the experimental group exhibited a shorter puncture time, required fewer needles, required needed fewer passages, and achieved a higher degree of conformity between the actual intraoperative target calyx and the planned preoperative target calyx (*p* < 0.05) (Table 2). Postoperatively, the experimental group had a higher stone clearance rate than the control group, and there were no significant differences in total operative time, decrease in hemoglobin, postoperative complications, and length of hospital stay between the groups (*p* > 0.05) (Table 2). According to the Sampaio classification system, 18 (56.25%) A-I, 5 (15.63%) A-II, 6 (18.75) B-I, and 3 (0.09%) B-II collecting systems were observed in the experimental group, with stone clearance rates of 88.89%, 80%, 83.3%, and 33.3%, respectively.

**Table 2:** Comparison of the perioperative data of both groups.

Parameters	Experimental group (*n* = 32)	Control group (*n* = 32)	*p*-value
Puncture location time (min)	4.64 (0.84)	5.80 (1.07)	<0.001
Number of channels (*n*)	1.5 (3)	2 (3)	<0.001
Number of first puncture channels (*n*)	1 (2)	2 (3)	<0.001
Target calyx consistency (%)	90.63%	65.63%	0.018
Total operative time (min)	138.97 (20.86)	147.22 (26.34)	0.170
Decrease in hemoglobin (g/L)	7.06 (±6.5)	10.06 (±6.33)	0.066
Stone-free rate (%)	87.50%	65.63%	0.039
Clavien-Dindo grade of complications (*n*)			0.215
0	26	22	
1	4	5	
2	2	4	
3	0	1	
4	0	0	
5	0	0	
Length of hospital stay (days)	8.5 (6)	8 (6)	0.764

## 4 Discussion

PCNL has a long learning curve. Studies have shown that practitioners need to perform 115 procedures to achieve excellent skills (12). Furthermore, the overall complication rate of PCNL is nearly 15%, and its rate of Clavien-Dindo class III or worse complications, such as interventional embolization, organ damage and sepsis is 5% (13). Improved stone clearance and reduced surgical risk are associated with the selection and establishment of percutaneous renal puncture pathways. Therefore, to fully understand the structural morphology of the renal pelvis and calyces, knowledge of the relationships between the stones, pelvis, calyces, and distribution of blood vessels, rational preoperative planning of the number and sequence of puncture channels, and accurate intraoperative nipple puncture are necessary to improve the stone clearance rate and reduce complications associated with complex renal stones (3). Researchers have attempted various methods to achieve this goal. Laser-guided puncture (14), visual puncture (15), flexible ureteroscopy-assisted puncture (16), iPad-based system tracking (Apple Inc., Cupertino, CA, USA) (17), electromagnetic tracking (18), robotic-assisted systems, and other assistive systems have improved the efficiency and accuracy of puncture procedures, correspondingly reduced the occurrence of postoperative complications (19, 20). Augmented reality, machine learning models, artificial intelligence(AI), and virtual reality technologies have also been used to improve simulation training, develop predictive models, and improve stone clearance, thus providing significant advantages when treating complex renal stones (21, 22). The increasing application of AI technology in urological surgery has improved clinical and surgical practices (23, 24). Particularly in surgical training, it reduces patient exposure, objectively evaluates surgical skills, and facilitates the creation of personalized teaching plans, its use in routine clinical practice has also driven medical advancements. However, due to the complexity and expense of these options, widespread promotion remains challenging.

Reportedly, 3D printing uses computer software to convert a two-dimensional image into a specific material (25). With the maturation of 3D reconstruction and fusion technologies, 3D engineering has garnered increasing attention from a expanding number of researchers in the medical field (26, 27), and it has been used to assist in preoperative surgical planning and surgical simulation due to its capacity to accurately print organs. The use of 3D reconstruction for complex renal tumors improves the understanding of the vascular anatomy, the surgical planning, and the prediction of surgical difficulty (28). Conventional two-dimensional information, such as that obtained from CT and ultrasound, does not provide complete anatomical images of tissues and local details and lacks the 3D morphological understanding of the renal pelvis and stones in relation to each other. Additionally, insufficient operator experience, poorly designed puncture channels, and inaccurate punctures of the target renal calyx are the major causes of complications (29). 3D printing technology has been applied to PCNL for complex kidney stones and horseshoe-shaped kidney stones because it can create 3D solid models of structures such as renal calyces, stones, blood vessels, and surrounding organs. These models can help physicians more intuitively consider the anatomical characteristics of the target area and interrelationships of the adjoining tissues and provide puncture guidance because of their accuracy. A well-designed puncture angle and pathway can reduce renal parenchymal tears caused by oscillating mirrors, thereby reducing the risk of other complications such as hemorrhage and renal artery embolism associated with multichannel puncture and improving stone clearance (30, 31). Preoperative simulation and training using 3D models enable quicker punctures during the procedure, thereby reducing puncture time and improving the success rate, this could simultaneously decrease the learning curve for novice physicians (32).

Although there are several nephrolithometric nomograms available to assess stone complexity, complications, and stone clearance rates, these outcomes are closely related to the morphology of the renal pelvis and calyces (33). Therefore, we utilized the Sampaio classification of the renal collecting system. Sampaio et al. categorized the renal collecting system into two types, A and B, according to the mode of drainage of the middle renal calyces (10). The type A collecting system (62.2%) includes two renal calyces, the upper pole and lower pole, through which the middle renal calyces drain urine. The calyces in the middle part of the kidney in a type A-I collecting system (45.0%) are subordinate to one of the major renal calyces in the upper or lower pole of the kidney, and the calyces in the middle part of the kidney in a type A-II collecting system (17.2%) are subordinate to the major renal calyces in the upper and lower poles of the kidney, respectively, with a calyx–neck crossover. The renal calyces in the kidneys that are not subordinate to the renal calyces in the upper and lower poles comprise type B collecting systems (37.8%). The type B-I (24.1%) collecting system includes the calyces in the middle of the kidney that drain urine to the renal pelvis through a separate large calyx. The type B-II (17.2%) collecting system includes a central part of the kidney that drains urine directly to the renal pelvis through two to four small calyces. Similar to the literature, this study found 18 (56.25%) type A-I, 5 (15.63%) type A-II, 6 (18.75%) type B-I, and 3 (9.38%) type B-II collecting systems in the affected kidneys in the experimental group using 3D reconstruction (total of 32 collecting systems). Additionally, we found that most of the staghorn stones in the type A-I collecting system occupied an enlarged renal pelvis and calyces, and that the necks of the middle calyces were wider, making it easier to reach all calyces by performing punctures in the upper calyx or middle and posterior calyces. The angle of oscillation of the mirror was larger, allowing for complete removal of the stone in a single channel; furthermore, the stone removal rate was the highest in the experimental group (17/18 patients). Most of the middle calyceal necks of type A-II collecting systems are elongated, and the middle anterior calyceal stones should be considered subordinate to the large calyces of the unperforated upper or lower kidney pole. Access mirrors do not reach the middle anterior group of calyces, making it difficult to complete the procedure through a single channel; therefore, it may be necessary to perform punctures in both upper and middle calyces to safely remove the stones. In B-I collecting systems, the necks of both the middle and upper calyces are elongated, resulting in low stone clearance; however, simultaneous punctures of both the upper and middle calyces can improve stone clearance. In our study, we found that the type B-II collecting system has two to four parallel calyces in the middle calyces of the kidney; therefore, it is not possible to completely clear the stone by performing a single pass of either the upper calyx or middle calyx due to the limited space in which the mirror can swing and reach. In the type B-II collecting system, three to four channels are commonly required for stone fragmentation, and the stone clearance rate is the lowest (1/3 patients). Although the B-II type of renal pelvis collecting system is less common, we believe it is the most complex. Therefore, we believe that 3D technology incorporating the Sampaio classification system for PCNL can help physicians fully understand the complex anatomy of the renal pelvic collecting system and its relationship with renal stones and the angles between the renal calyces by providing them with more detailed anatomical information that allows for a reasonable design of the number of puncture channels and puncture sequence, predict the stone clearance rate, formulate a reasonable surgical plan, optimize the puncture process during surgery, and improve the stone clearance rate, thus reducing the surgical difficulty caused by the diversity and complexity of the renal collecting system.

## 5 Study limitations

This study had some limitations. First, it was a nonrandomized retrospective study performed at a single center with a limited number of cases; therefore, the statistical analysis of the data may have been biased. Our results should be confirmed by a multicenter, randomized, controlled study with a large sample size.

## 6 Conclusions

3D technology integrated with Sampaio's theory of ensemble system typing is a novel and effective approach to managing complex renal stones. This method reduces the puncture time, improves puncture success, reduces the number of channels, and improves stone clearance. Additionally, this method enhances the subjective 3D experience of surgery, thereby providing an objective, accurate, and reliable reference for surgical planning and specific implementation of programs involving PCNL.

## Data Availability

The original contributions presented in the study are included in the article/Supplementary Material, further inquiries can be directed to the corresponding author.
